# Supplementary material for: Molecular Hydrogen for Outpatients with COVID-19 (Hydro-COVID): A Phase 3 Randomised, Triple-Blinded, Pragmatic, Placebo-Controlled, Multicentre Trial
Source: J Clin Med. 2024 Jul 24;13(15):4308. doi: 10.3390/jcm13154308 (PMC11313273; doi:10.3390/jcm13154308)
Supplement: Supplementary file 1 [file jcm-13-04308-s001.zip › jcm-3080534-supplementary.pdf]

Hydro-Covid : Supplementary materials

Material and methods

Study design

A phase 3 double-blinded, parallel-group, randomised, placebo-controlled trial (RCT) evaluating the safety and efficacy of H2 for COVID-19 disease in adult outpatients was initiated on January, 2021. Outpatients with mild COVID-19, according to the World Health Organisation (WHO) guidelines, and with at least one risk factor, were eligible, regardless of whether they had been vaccinated against SARS-CoV-2.

This trial was conducted in 5 French and 1 Serbian region. It was coordinated by the TIMC public laboratory (Grenoble, France). The trial was done in accordance with the principles of the International Conference on Harmonisation of Good Clinical Practice Guidelines.

Participants

Partnerships were organised with nasopharyngeal swabbing teams and laboratories analysing SARS-CoV-2 retro-transcription polymerase chain reaction (RT-PCR), general practitioners offices, home-care nurses, pharmacies, and vaccination centres. Several community outreach strategies were used, including physical (posters, leaflets, and newspapers) and social (radio, network) media to publicise the trial. At the beginning of the study, through communication tools or encouragement from their health professionals, patients were invited to call the investigation centre to propose their participation. Four months later, due to a low inclusion rate, French medical analysis laboratories in the recruitment area were invited to contact by telephone each patient who tested positive for COVID-19 PCR. If the patient expressed interest, the trial team was informed, and, in turn provided information to the patient and proceeded with pre-inclusion. An inclusion visit by video teleconsultation, or, alternatively, by telephone with a trained physician investigator was arranged, the data were recorded in an electronic case report form (CRF). Products delivery and paper CRF were carried out at the patient's home immediately after its inclusion by research team, a medical doctor or a home care nurse partner. Oxygen status was controlled at home before product delivery with a pulse oximeter (superior or equal to 95% to be included) and left at the disposal of the patient to complete the paper CRF during follow-up. In order to increase the inclusion rate, which has been slowed down by the different epidemic waves, Serbia has been associated with this RCT. Eligible Serbian patients started to be screened in May 2021. Due to the differences in health organisations in France and Serbia, the inclusion and product delivery were carried out in Serbia directly by the investigators during the medical visit.

Inclusion criteria at randomization were as follows: SARS-CoV-2 is biologically confirmed for less than 4 days by antigen test or RT-PCR, onset of signs or symptoms for less than 4 days earlier, at least one sign or symptom of COVID-19, and at least one risk factor for development of severe COVID-19 (age >60 years; treated hypertension; obesity; all types of treated diabetes; serious heart conditions [heart failure, coronary artery disease, or atrial fibrillation; history of stroke; stage 3 chronic renal failure [30 ⩽ estimated GFR <60 mL / min

/ 1.73 m^2]; chronic obstructive pulmonary disease, including chronic respiratory failure under long-term oxygen therapy; active cancer or one diagnosed in the last 5 years; immunodeficiency of therapeutic origin or HIV infection and last known CD4 count <200 /mm^3; history of pulmonary embolism and/or proximal deep vein thrombosis; asthma under inhaled corticosteroid therapy; paired sleep apnea syndrome; peripheral arterial disease of the lower limbs, stage II and above; another risk factor presented, according to the list defined by the French High Council of Public Health.43 patients with three minor cumulative conditions (i.e., overweight, social vulnerabilities, hypothyroidism, immune, or not listed inflammatory disease) could also be eligible, according to the investigator. At the beginning, of the epidemic, many patients were frustrated by the lack of access to biological tests, as the sampling laboratories were saturated. In the absence of biologically confirmed nasopharyngeal swab, a patient was eligible if he or she presented at least three clinical signs among 11 (fever > 37.5°C for at least 3 days; cough; sore throat/cold; headache; anosmia, dysgeusia; myalgias, arthralgias, bone pain; breathing difficulties [feeling of dyspnea at rest]; chest pain [sternal]; digestive complaints [diarrhoea, nausea, vomiting]; tachycardia [palpitation]; conjunctivitis [red eyes]), and notion of contact (with a COVID+ certain or probable patient) within the last 10 days. In this situation, patients could be included under the condition to obtain a positive result during the following days.

Exclusion criteria were a negative RT-PCR test, absence of an attending or referring physician, oxygen saturation < 95% at baseline, any sign of seriousness incompatible with home care, severe chronic renal failure or requiring dialysis (i.e., eGFR <30), uncontrolled clinically significant heart disease, pregnancy, or patient under guardianship or curatorship. Any treatment necessary for the management of acute or chronic patient conditions was allowed, (including, for example, any monoclonal antibody, anti-viral drugs, vitamin D, ivermectin, zinc, colchicine, …). All subjects were asked to maintain the same lifestyle throughout the study. Written informed consent was obtained from all patients.

Randomisation and masking

Baseline data was collected using an online case-report form that included demographics, major comorbidities, any treatment or dietary supplement taken by enrolled patients during the study. Eligible and consenting subjects were then randomized in a double-blind fashion to either the intervention (HRW) or placebo group by computer-generated random numbers in a 1:1 ratio. Randomisation was stratified in blocks of four stratified by age (< 70 or ⩾ 70 years).

Hydro-Covid was initiated on January 22, 2021, when the first participant was screened. The last participant was enrolled between on March 24, 2022, and completed the one-month visit between on April 25, 2022. The trial team, investigators, and participants are not informed of treatment allocation until all participants have completed the one-year follow-up visit. The HRW pill and the placebo were packaged in identically shaped bottles. An unblinding procedure has been foreseen in cases of necessity (severe allergy, life-threatening

emergency) at the request of any physician and validated by the trial team.

Interventions

All participants received the usual standard of care for COVID-19 provided by their general practitioners. High-concentration HRW was prepared via H2 -producing tablets (Drink HRW), donated by HRW Natural Health Products Inc. (New Westminster, BC, Canada). H_2 is produced by the active ingredient, metallic magnesium (80 mg), which reacts with water to produce H_2 gas and magnesium ions, according to the reaction: $Mg + 2H_2O \rightarrow H_2 (g) + Mg^{++} + 2(OH)^-$. By dissolving a tablet in a 250-mL cup, 3.3 moles of H2 are produced. A supersaturated solution is obtained during the first 30 minutes, with a concentration of about 4 moles/L of H2. Participants consumed 1 tablet twice daily in 250 mL of 12-18°C water. They were advised to drink the product in one gulp as soon as the tablet finished dissolving on an empty stomach. Therefore, each patient received each ingested tablet around 1 millimole (2 mg) of H_2.
Hydrogen and placebo appeared identical. Placebo, also donated by HRW Natural Health Products Inc. (New Westminster, BC, Canada) contained identical ingredients to the hydrogen supplement, but instead of metallic magnesium, the placebo contained magnesium carbonate.

Socio-demographic characteristics and comorbidities were collected at baseline. The quality of life was measured by the EuroQol 5 dimensions (EQ5D-5L), and the quality of sleep by the Pittsburgh Sleep Quality Index (PSQI). Fatigue was approached by the Chalder scale and breathlessness by the mMRC (modified Medical Research Council). Data were collected every day during the first month on a paper CRF at home by participants, including HRW absorbed per day or changes in the severity of the main symptoms measured by visual analogic scale (VAS) self-assessment. Oxygen status was assessed by means of a pulse oximeter for non-invasive arterial oxygen saturation and pulse.
The primary outcome was collected at Day 12-14 by video teleconsultation, phone, or during medical visit by physician investigators, according to the health organisation of the 2 countries in the face of the pandemic. Secondary outcomes were collected at 1, 3, and 12 months by postal questionnaire and phone calls by the research team. All adverse events were reported. Reportable adverse events included serious adverse events, adverse events resulting in study medication discontinuation, and adverse events assessed as possibly related to study medication.

Outcomes
The primary endpoint is a composite endpoint of symptom worsening (dyspnea and fatigue), O2 loading at home or in an emergency room, hospitalisation (not only a need for the emergency service) and death occurring within 14 days of inclusion in the study. Worsening of fatigue was defined by a 25% increase via the Chalder scale (i.e., an increase $\geqslant$ 5 points for physical symptoms and $\geqslant$ 3 points for mental symptoms) or via daily VAS self-assessment for fatigue (i.e., an increase $\geqslant$ 25 points). Worsening of dyspnoea was defined by a 25% increase

of the mMRC scale (i.e. an increase ⩾ 1 point if mMRC at baseline ⩾ 1 or an increase ⩾ 2 points if mMRC at baseline = 0) or a daily VAS self-assessment (i.e. an increase ⩾ 2 5 points). Secondary outcomes included time to clinical improvement and the number of days with dyspnea, or fatigue, time to hospitalisation for any cause or due to COVID-19 progression, all-cause mortality and time to death from any cause, quality of life and quality of sleep, adverse reactions to the study medications, and the proportion of participants who are nonadherent with the study. All secondary outcomes were assessed up to one month following randomization.

Safety

Safety endpoints included adverse events occurring during the treatment period (from day 28 or earlier), serious adverse events, and adverse events resulting in discontinuation of treatment or placebo. Incidence data were provided for each treatment group, including all patients who received at least one dose of H_2 or placebo. The investigators actively collected safety information from the time of informed consent through day 28. Severity of adverse events were determined according to the International Conference on Harmonisation (ICH) guideline ICH E2A[44].

Statistical analysis

The primary analysis compared the proportions of patients in the two groups, measuring the efficacy of H2 compared with placebo. It was assessed by the first primary efficacy end point recording up to day 14, using the Kaplan-Meier method to account for all patients, including those prematurely withdrawn from the trial or lost to follow-up. The adjusted hazard ratio was calculated, stratified by age 70, and associated with 90 confidence intervals for analysis using Cox proportional model. Secondary outcomes were classically analysed by student or Mann-Whitney test for qualitative variables, chi2 or Fisher test for quantitative variables. Quality of life according to the EQ5D5L will be measured by analysing the area under the ROC curve. The planned enrollment of 700 participants was selected to ensure greater than 95% power to demonstrate superiority at the primary end point at a one-sided 2.5% alpha level if the underlying event rates were 24% with HRW and 32% with placebo. All analyses were conducted with the use of STATA, version 16 (STATA corp., LLC).

A prespecified interim analysis for early efficacy, futility, and safety was planned to occur once the first hundred participants had been enrolled (achieved on July 21, 2021). This analysis was based on the primary efficacy end point according to prospectively set stopping boundaries that were reviewed by regulatory agencies.
